# Research advances of pan-immune inflammation value in rheumatoid arthritis

**DOI:** 10.3389/fimmu.2025.1693676

**Published:** 2025-11-19

**Authors:** Chengmei Zhou, Xinyu Cheng, Junjie Zhang

**Affiliations:** 1 Department of General Practice, Central Hospital Affiliated to Shenyang Medical College, Shenyang, Liaoning, China; 2 Department of Emergency, Shenyang Heping District Beishi Community Health Service Center, Shenyang, Liaoning, China; 3 Department of Pathology, Central Hospital Affiliated to Shenyang Medical College, Shenyang, Liaoning, China

**Keywords:** rheumatoid arthritis pannus, immune cells, cytokines, peripheral blood, pan-immune inflammation value

## Abstract

Rheumatoid arthritis (RA) is a persistent autoimmune condition marked by systemic inflammation, primarily impacting multiple joints and causing irreversible structural and functional damage. While pathophysiology remains complex, immune cells and cytokines are crucial to the disease’s initiation and progression. C-reactive protein (CRP) and erythrocyte sedimentation rate (ESR) are routinely used to assess disease activity. However, in recent years, the pan-immune inflammatory value (PIV) has emerged as a focal point of inquiry. As a new, single biomarker calculated from various routine blood parameters, PIV accurately reflects the patient’s comprehensive systemic immune-inflammatory condition and offers a more integrated perspective than traditional markers. This article provides a focused analysis of the potential role and clinical significance of PIV in assessing the activity and advancement of RA.

## 1 Introduction

Rheumatoid arthritis (RA) is an autoimmune disorder generally defined by chronic, progressive, and erosive arthritis, exhibiting symmetry and affecting many joints (1). In the initial phase of the disease, symptoms including pain, edema, and morning stiffness predominantly impact tiny joints, such as those in the hands and feet, with morning stiffness frequently lasting over one hour (2). As the disorder advances, major joints become damaged; in the later stages, even extra-articular tissues, including the skin, lungs, heart, and blood vessels, may be compromised, significantly diminishing patients’ quality of life (3).

While the pathophysiology of RA remains incompletely understood, extensive research has established that immune cells and cytokines are crucial to the advancement of RA (4). Abnormal immune system activation leads to atypical proliferation of synovial tissue within joints. Excessive proliferation of synovial cells, extensive infiltration of inflammatory cells (including lymphocytes and macrophages), and intense neovascularization collectively form an erosive tissue known as pannus (5). The pannus secretes proteases that destroy the cartilage matrix, hence compromising the normal joint structure (6). This finally results in joint space constriction, bone degradation, and potential loss of function, which constitutes the primary detrimental mechanism accountable for disability in RA (7).

The aberrant activation of immune cells, coupled with the cytokine “network amplification effect,” is essential (8). Reactive T cells, specifically Th1 and Th17 subsets, stimulate immune cells such B cells and macrophages, resulting in their aggregation within the joint synovium (9). B cells exhibit hyperproliferation and generate autoantibodies, including rheumatoid factors, which create immune complexes that accumulate in the joints, so exacerbating inflammatory responses (10).

Macrophages release essential cytokines, including TNF-α, IL-6, and IL-1, which directly promote the sustained proliferation of synovial cells (11). This intensifies local inflammatory symptoms, including erythema, edema, hyperthermia, and discomfort, while also stimulating osteoclasts through signal transduction, thereby accelerating bone degradation and loss. It concurrently hinders the reparative function of osteoblasts, creating a harmful cycle of “destruction exceeding repair,” which ultimately results in joint structures transitioning from inflammatory damage to irreversible deformity and functional impairment (12).

Historically, clinical practice frequently depended on the measurement of CRP and ESR in patients’ peripheral blood to evaluate the status of RA (13). In recent years, numerous cell ratio indicators in standard blood tests have been extensively utilized in RA research as supplementary markers for illness assessment. The PIV, originating from whole blood indicators and characterized by ease of use and robust practicality, has emerged as a focal point of research among scholars in recent years (14). Literature indicates that these indicators demonstrate potential in forecasting the prognosis of pediatric disorders linked to immunological deficiencies (15). In several rheumatic disorders, including RA, familial Mediterranean fever, and vasculitis, these indicators have demonstrated efficacy in monitoring disease activity. They are also significant in forecasting tumor prognosis and therapy effectiveness (16). This review elucidates the role of PIV in the course of RA, with the objective of enhancing RA monitoring and aiding its clinical management.

## 2 The correlation between PIV and rheumatoid arthritis

The pathophysiology of RA is intricate, with immunological system disorders being a significant factor in its initiation (17). Dysregulation of cellular and humoral immunity, coupled with disturbances in the inflammatory cytokine and chemokine network, consistently infiltrates joint synovial tissue, resulting in the degradation of cartilage and bone (18).

### 2.1 B lymphocytes

B cells and T cells are fundamental components of the immune system. In RA, considerable stimulation by inflammatory agents leads to the hyperactivation of B cells, which generate substantial amounts of IgM antibodies, referred to as rheumatoid factor (RF) (19). These antibodies identify the Fc region of immunoglobulins, creating immune complexes that accumulate in the joint synovium, thereby activating complement and inflammatory cells (including macrophages and neutrophils), and releasing significant hydrolytic proteases that harm joint tissues (20).

Interleukin-6 (IL-6), a B-cell differentiation factor, promotes the conversion of B cells into plasma cells for antibody synthesis upon external stimulation; it also induces the secretion of the pro-inflammatory cytokines tumor necrosis factor-alpha (TNF-α) and IL-6. In RA, interleukin-6 is frequently overexpressed. The aberrant activation and differentiation of B cells result in heightened autoantibody synthesis, hence intensifying autoimmune reactions, osteoporosis, and cartilage tissue damage. Consequently, therapeutic approaches aimed against IL-6 have emerged as a prominent focus in the treatment of these disorders (21).

In RA, the hyperactivation of B lymphocytes causes the depletion of T lymphocytes, leading to immunological dysfunction. Activated T cell production transpires when B cells, through their surface B cell receptors (BCRs), identify and internalize autoantigens present in the joint, such as citrullinated synovial proteins (22). Following processing, these antigens are presented to CD4+ T cells through MHC-II molecules, thereby activating the T cells. The excessive activation of B lymphocytes leads to considerable depletion of T cells that secrete large quantities of pro-inflammatory cytokines, such as IL-6, TNF-α, and IL-17. Excessive pro-inflammatory cytokines create a chronic inflammatory microenvironment, accelerating T cell activation and depletion. Specific cytokines, such as IL-10 (23) may indirectly diminish the quantity of functional T cells by suppressing T cell growth or triggering death.

### 2.2 T lymphocytes

Recent investigations have demonstrated that autoreactive T cells are crucial in the pathogenic advancement of RA. Th17 cells emit the cytokine IL-17, which prompts epithelial cells, endothelial cells, and fibroblasts to generate numerous chemokines, including CXCL8 (IL-8) (24), thus enlisting neutrophils to the locus of infection to perform immunological phagocytic functions. It can stimulate the activation and multiplication of T cells and B cells, while augmenting the phagocytic and bactericidal abilities of macrophages (25). It can interact with other cytokines to form a complex cytokine network, collectively influencing the intensity and characteristics of immune responses. A moderate concentration of IL-17 improves immune defense, whereas elevated IL-17 levels can induce immunopathological reactions.

In RA patients, IL-17 levels in synovial tissue, synovial fluid, and peripheral blood are markedly elevated, frequently correlating with increased joint swelling, heightened pain scores, and elevated inflammatory markers such as CRP and ESR, signifying its role in the inflammatory processes of RA (26). Consequently, identifying pertinent indications of reactive T cells aids in the diagnosis and surveillance of autoimmune disease progression.

### 2.3 Macrophages

Macrophage progenitor cells are monocytes that, when activated by external stimuli such as infections and rheumatoid factors, secrete substantial amounts of TNF-α, IL-6, interleukin-1 (IL-1), and various other cytokines. These substances facilitate the recruitment and assembly of neutrophils (11), activate T and B cells, and establish a “inflammatory network,” resulting in the sustained aggravation of joint synovial inflammation.

Chronic inflammation induces the proliferation of synovial cells, culminating in the development of “pannus” (5) (granulation tissue produced by synovial hyperplasia) that exacerbates joint erosion, ultimately causing joint deformity and functional impairment. Activated macrophages can produce enzymes, including matrix metalloproteinases (MMPs) (27), which can directly harm articular cartilage and bone tissue.

In patients with RA, TNF-α is produced in significant quantities by macrophages and T cells in reaction to heightened external stimuli. TNF-α induces inflammatory responses, leading to synovial hyperplasia and edema, accompanied by symptoms such as joint erythema, swelling, pain, and stiffness. TNF-α induces the activation of synovial cells and osteoclasts, accelerating the degradation of articular cartilage and bone tissue (28). Ultimately, this may result in joint deformity and functional impairment, rendering it a significant contributor to the disability associated with RA. TNF-α stimulates the production of additional inflammatory mediators (such as IL-6 and IL-1), creating a “inflammatory cascade” that intensifies the immune systems assault against the body’s own joint tissues.

### 2.4 Neutrophils

During the initial phase of RA pathogenesis, neutrophils are activated by inflammatory mediators in the synovium, including TNF-α and IL-1, leading to their accumulation in the joint synovium and synovial fluid, which subsequently instigates localized inflammatory responses characterized by joint erythema, edema, and pain (29). Activated neutrophils secrete cytokines (e.g., IL-8 and TNF-α) and chemokines, which subsequently attract other immune cells (e.g., monocytes and T cells) to the synovium and synovial fluid, therefore intensifying the inflammatory response.

IL-8 (interleukin-8) is a potent neutrophil chemokine that facilitates the recruitment of neutrophils from peripheral blood to the joint synovium and synovial fluid, thereby playing a critical role in the initiation of inflammatory responses. Neutrophils, attracted by IL-8, generate additional inflammatory mediators such as TNF-α and IL-1 upon activation, exacerbating synovial inflammation and tissue damage. IL-8 directly induces the proliferation of synovial fibroblasts, compromises the cartilage matrix and synovial blood vessels, and accelerates the progression of joint deformity and functional impairment (30).

### 2.5 Platelets

Platelets exhibit major histocompatibility complex class II molecules (MHC-II) on their surface, facilitating antigen presentation to activate T cells and modulating immunological responses. Aberrant immune activation in RA signifies a crucial link in the disease’s development and progression. Platelets can synthesize and secrete cytokines such as IL-1, IL-6, and TNF-α (31), which facilitate the recruitment and activation of inflammatory cells, hence amplifying the inflammatory response.

Platelets secrete vascular endothelial growth factor (VEGF), which promotes the development of vascular endothelial cells, thereby facilitating the infiltration of inflammatory cells and exacerbating joint inflammation and injury.

### 2.6 PIV

A notable presence of lymphocytes, macrophages, neutrophils, and platelets is evident in the peripheral blood of individuals with rheumatoid arthritis. Inflammatory biomarkers such as C-reactive protein (CRP) and erythrocyte sedimentation rate (ESR) (32) are routinely utilized in clinical practice to diagnose and monitor disease activity in RA, notwithstanding their limited sensitivity and specificity. In recent years, comprehensive blood cell component testing has been utilized to evaluate systemic inflammation in both rheumatic and non-rheumatic disorders, such the systemic immune-inflammation index (SII) and PIV. The SII is computed as (neutrophil count × platelet count)/lymphocyte count, facilitating a superior evaluation of RA compared to the previously described individual indicators (33).

The PIV, initially introduced in 2020, is a biomarker derived from total blood cell count that monitors immune-inflammatory activity. The formula for calculating the PIV (33) is (neutrophil count × platelet count × monocyte count)/lymphocyte count. The PIV incorporates the monocyte count into its formula alongside the components of SII, facilitating a more accurate depiction of the synergistic effects of immune cells in chronic inflammation. An elevated PIV indicates an increase in neutrophils, platelets, and monocytes, accompanied by a decrease in lymphocyte count, potentially reflecting a significant inflammatory response. The PIV indicates a synthesis of various markers related to inflammation and the immune system, enabling effective detection of changes in immunological and inflammatory parameters commonly observed in RA. Compared to previous measures such as CRP, ESR, and SII, it provides a more comprehensive indication of immunity and inflammation levels in RA, serving as an important tool for evaluating RA staging and monitoring treatment responses.

The comprehensive blood parameters utilized to calculate the PIV are easily accessible and extremely practical, rendering it a focal point of research among researchers in recent years. Literature indicates that it has demonstrated potential in forecasting the prognosis of pediatric disorders linked to immunological abnormalities. In several rheumatic disorders (including RA, familial Mediterranean fever, and vasculitis) (34), it has been demonstrated to be a helpful marker for assessing disease activity. It is significant in forecasting tumor prognosis and treatment effectiveness.

## 3 Application of PIV in RA

The interaction of immune system function, physiological stress reactions, and treatment measures frequently leads to cycles of remission and progression (35) in the course of RA. During the progression phase, the hyperactive immune system targets joint tissues, leading to increased inflammation and further joint deterioration.

RA is clinically evaluated through the presence of pain and swelling in the tiny joints of the hands and feet, morning stiffness, and the Disease Activity Score 28 (DAS28) (36). The DAS28 is computed by assessing the count of 28 tender joints, 28 swollen joints, erythrocyte sedimentation rate, and the patient’s overall evaluation using a visual analog scale. It measures disease activity in RA, is crucial in daily therapy, and has received endorsement from the European League Against Rheumatism (EULAR) (36) and the American College of Rheumatology (ACR).

A DAS28 score greater than 5.1 indicates increased disease activity, requiring timely medical intervention or treatment adjustment. A score between 3.2 and 5.1 indicates moderate disease activity, requiring timely and ongoing monitoring and management. A score ranging from 2.6 to 3.2 indicates moderate disease activity, suggesting that conditions are well-managed, although ongoing care remains essential. A score below 2.6 indicates disease remission, marked by the absence of active disease, mild symptoms, and a stable health status (36).

In June 2024, scholar Ipek Okutan performed a retrospective study on RA (37). This was the inaugural assessment of the diagnostic and prognostic efficacy of PIV, SII, and the Systemic Inflammation Response Index (SIRI) in RA. The study included 97 RA patients, evaluated for disease severity using DAS28 (64 in the active phase and 33 in remission), with 51 healthy controls. Compared to the control group, the 97 RA patients exhibited significantly elevated metrics such as white blood cell (WBC) count, neutrophil count, monocyte count, platelet count, ESR, CRP, SII, PIV, and SIRI, while their lymphocyte counts were notably decreased. This indicates that the aforementioned blood signs can be recognized as markers for the identification of RA.

This research analyzed the relationships among SII, PIV, and SIRI in patients during the active phase, those in remission, and healthy controls. The SII readings in RA patients during the active phase were higher than those observed in remission and in healthy controls. An elevated SII value is associated with a heightened chance of developing active-phase RA. Comparable results were also documented by Liu et al (38). To provide a clearer context for these emerging systemic inflammatory markers, we present a comparison of the formulas, components, and general clinical utility of PIV, SII, and SIRI in **Table 1**.

**Table 1:** Comprehensive comparison of PIV, SII, and SIRI in RA.

Index	Components of blood cells	Formula	Clinical utility in RA
PIV	N, P, M, L	PIV = N × P× M/L	Disease Activity Assessment: High PIV is positively correlated with the Disease Activity Score 28 (DAS28), suggesting heightened disease activity and inflammation. Prognosis/Monitoring: May serve as a complementary marker for diagnosis and monitoring treatment efficacy.
SII	N, P, L	SII = P × N/L​	Disease Activity Assessment: Elevated SII is positively correlated with DAS28 scores. Therapeutic Prediction: Reported to be among the strongest predictors of the efficacy of TNF-α inhibitors (a class of biological drugs) in RA patients.
SIRI	N, M, L	SIRI = N × M/L​	Disease Activity Assessment: Elevated SIRI shows a positive correlation with the DAS28 score. Monitoring: Similar to PIV and SII, it is used as a reflection of the overall inflammatory burden in RA patients.

RA, Rheumatoid arthritis; PIV, Pan-Immune-Inflammation Value; SII, Systemic Immune-Inflammation Index; SIRI, Systemic Inflammation Response Index; N, Neutrophils; P, Platelets; M, monocytes; L, Lymphocytes.

The study conclusively proved that PIV may effectively differentiate between RA patients in remission and healthy controls, achieving statistical significance. A literature review indicated that just one study (39) has assessed the correlation between PIV and RA, yielding results that align with theirs.

The study showed that the concentrations of the specified blood indicators, PIV, SII, etc., are markedly increased in RA, implying that these markers can effectively track the evolution of RA. As RA disease activity escalates, the levels of PIV and SII markedly increase, indicating that PIV and SII may function as inflammatory biomarkers for assessing disease activity in RA patients. In the comparison of PIV with SII, PIV correlates with disease activity, accurately differentiates between RA patients in remission and healthy controls, functions as a measure for evaluating RA disease activity, and assists in monitoring treatment effectiveness and enhancing patient prognosis.

In 2024, Dr. Pınar Özge Başaran executed the inaugural study comparing the levels of PIV and SII in patients with RA (33). The research indicated that, in contrast to SII, PIV correlates with illness activity. It may accurately distinguish between RA patients in remission and healthy controls, serving as a marker to assess RA disease activity. PIV is advantageous for assessing therapy outcomes and enhancing patient prognosis. The study included 116 participants, comprising 67 patients with diagnosed RA, whose disease severity was evaluated by DAS28—35 in the active phase and 32 in remission—and 49 control people.

The experimental results indicated that neutrophil levels in the active-phase RA group were considerably elevated compared to both the remission-phase RA group and the control group (P < 0.001). The lymphocyte levels in the active-phase RA group were markedly lower than those in the other two groups, with statistical significance (P < 0.001). Monocyte levels in both the active-phase and remission-phase RA groups were statistically substantially elevated compared to the control group (P < 0.001).

Statistically significant disparities were noted in CRP, PIV, and SII levels across the groups. The CRP levels in the active-phase RA group were considerably elevated compared to the remission-phase group and the control group (P < 0.001). The PIV and SII levels in the active-phase RA group were significantly elevated compared to the remission-phase group and the control group (P < 0.001). Additionally, the PIV and SII levels in the remission-phase group were also significantly higher than those in the control group (P < 0.001). These findings align with the data presented by scholar Ipek Okutan (38).

This research is the inaugural investigation of the correlations among PIV, SII, and CRP. Assessments were performed on individuals with active-phase RA, individuals with remission-phase RA, and healthy controls. Furthermore, the sensitivity and specificity of inflammatory measures within each group, along with their respective advantages, were examined.

PIV and SII are straightforward, cost-effective, and dependable indicators for forecasting remission in RA patients. In comparison to the control group, no significant difference in CRP levels was noted among RA patients in remission; nevertheless, substantial changes were observed in PIV and SII levels. Furthermore, in the RA remission cohort, the sensitivity and specificity of PIV surpassed those of SII. In RA patients exhibiting elevated disease activity, C-reactive protein (CRP), PIV, and SII are all effective indicators of disease activity as compared to healthy controls and RA patients in remission.

In 2023, researcher Duygu Tutan performed a retrospective study to examine the correlation between PIV and RA (15). The study elucidates the essential elements of RA, encompassing its disease features and pathophysiology. It elucidates the function of cellular inflammatory mediators and autoreactive T lymphocytes in the pathological advancement of RA. This study delineates the current research state of PIV as an inflammatory evaluation marker in various tumor illnesses and other conditions, with the objective of investigating the association between RA and PIV.

The study enrolled 64 patients with RA and 40 healthy control participants. The study included values of neutrophils, lymphocytes, platelets, monocytes, and red blood cell distribution width (RDW) obtained from standard hematological studies in both the patient and control cohorts. Subsequently, comparison analyses were conducted to assess immune-inflammatory markers between the two groups. Additionally, these indicators were categorized and analyzed according to the DAS28 activity levels within the patient group.

A primary benefit of PIV is that its constituent parameters are often assessed indices in RA, with these assessments being economical and simple to execute. This study demonstrates that PIV serves as an effective marker for differentiating RA patients from healthy individuals, evaluating remission and active stages of the disease, and monitoring disease activity in patients with active RA.

This research delineates the illness features, progression, and the utilization of the DAS28 score in assessing disease activity in RA. It elucidates the function of cellular inflammatory mediators and autoreactive T lymphocytes in the pathogenic advancement of RA. This document outlines the contemporary research state of PIV, an innovative marker for inflammatory evaluation in recent years, across many tumor illnesses and autoimmune disorders.

The three previously mentioned papers assessed the prediction accuracy of the PIV and SII parameters by Receiver Operating Characteristic (ROC) analysis. Precise prognostic markers are essential for the diagnosis and evaluation of RA. Researchers examined the characteristics of PLR and SII within defined periods to ascertain their sensitivity and specificity in diagnosing RA. Sensitivity indicates a parameter’s proficiency in identifying persons with the disease, whereas specificity illustrates its effectiveness in correctly recognizing those without the ailment.

The ROC curve intuitively illustrates the variations in sensitivity and specificity of PLR and SII across multiple thresholds, facilitating the identification of the best diagnostic threshold. This offers quantitative evidence for doctors in assessing RA, aids in diagnostic decision-making, and promotes early detection and therapy for patients.

This publication references research by scientists Ipek Okutan and Pınar Özge Başaran from 2023 to 2024, which examined the correlations of PIV and SII with the active and remission phases of RA, as well as healthy control groups. PIV is interpreted as a blood index ratio that amalgamates many signs of inflammation and the immune system. It can detect inflammatory alterations in immunological and inflammatory markers typically seen in RA, making it a potentially significant instrument for evaluating systemic inflammation levels in RA.

## 4 The role of PIV in rheumatoid arthritis

RA is an autoimmune disorder. The fluctuation between its active and remission phases is chiefly linked to the interaction of aberrant cellular immunity and cytokine dysregulation. This creates a detrimental loop of “immune cell activation → cytokine release → inflammation exacerbation → additional immune cell activation.” This results in synovial inflammation and bone degradation, presenting as active-phase symptoms including joint swelling, pain, and stiffness. When inflammation is temporarily suppressed, the condition enters a state of remission.

Consequently, biological therapies aimed at certain cytokines have been created clinically. These medicines stop the vicious cycle by inhibiting their effects.

Anti-TNF-α drugs (e.g., etanercept, adalimumab) and anti-IL-6 receptor medications (e.g., tocilizumab) (40). These medications proficiently regulate inflammation, diminish joint damage, and assist in the transition of patients from the active period to remission.

Blood routine ratios, serving as supplementary markers for illness evaluation, have been utilized in studies concerning RA. Clinically, efforts have been undertaken to indirectly indicate inflammatory activity by cellular ratios in blood analyses. \The neutrophil-to-lymphocyte ratio (NLR) signifies an active phase when elevated, resulting from heightened neutrophil levels (inflammatory response) and diminished lymphocyte counts (immune suppression). Monocyte-to-lymphocyte ratio (MLR): The increase in this ratio may correlate with inflammatory activity due to the role of monocytes in the release of inflammatory factors. Platelet-to-lymphocyte ratio (PLR) (41): Considering that platelets may rise during inflammation, an increase in this ratio could indicate disease activity.

These ratios, noted for their operational simplicity and cost-effectiveness, can function as supplementary markers for differentiating between the active and remission stages of RA. Nonetheless, their specificity is constrained, necessitating thorough assessment alongside clinical manifestations, inflammatory indicators (e.g., CRP, erythrocyte sedimentation rate), and imaging studies.

Identifying the blood parameters that most efficiently diagnose disease activity in RA and evaluate therapy responses is critically important. Considering the advancement of more extensive calculating methods for immune cells, including PIV, SII, and SIRI.

SII is a biomarker obtained from computations based on comprehensive blood cell counts (42). This count, which includes neutrophils, platelets, and lymphocytes, is utilized to evaluate inflammatory levels. Increased SII values arise when neutrophil and platelet counts are elevated, accompanied by diminished lymphocyte counts, signifying a vigorous inflammatory response 11. SII has been assessed in psoriatic arthritis, lupus, and RA, revealing correlations between SII and disease activity levels (37, 38, 43).

SIRI is a biomarker calculated from the ratios of neutrophils and monocytes to lymphocytes. Prior research has demonstrated that SIRI, as a significant biomarker, contributes to the onset and advancement of multiple malignant cancers (44).

Erre et al. (45) recently observed that SIRI can more comprehensively indicate persistent inflammation in rheumatic disorders, extending beyond RA, as evidenced by their research.

PIV, initially delineated by Fuca et al. (46), is employed to evaluate inflammatory state. PIV is determined by analyzing four categories of blood cell counts: neutrophils, platelets, monocytes, and lymphocytes. PIV has been studied in various rheumatic disorders, including RA, familial Mediterranean fever, and vasculitis.

The detrimental interplay between cellular immunity and cytokines is the fundamental mechanism driving the variable progression of RA (47–49). Simultaneously, tailored medicines and assessments of inflammation-related ratios offer essential instruments for the management of RA from the viewpoints of treatment and evaluation, respectively. Recently, PIV, a focal point of research, thoroughly assesses diverse blood indices and demonstrates potential as a beneficial marker for tracking the onset and advancement of RA. While our analysis highlights the potential of PIV as a cost-effective and integrated biomarker for assessing systemic inflammation in RA, several limitations inherent to the existing literature must be critically considered. A major drawback is that the majority of current studies examining PIV in RA are retrospective or cross-sectional in nature, which significantly limits their capacity to establish causal relationships (50), long-term prognostic value, or the utility of PIV in predicting therapeutic response over time. Furthermore, a challenge for clinical application is the observed heterogeneity and lack of consensus on standardized PIV cut-off values across different studies for defining RA disease activity or predicting outcomes. Consequently, there is an urgent need for large-scale, multi-center, prospective trials to rigorously validate PIV’s performance, establish standardized thresholds, and directly compare its longitudinal changes against established clinical metrics (e.g., DAS28, CDAI) and gold-standard inflammatory markers. Future research should also focus on the value of serial PIV measurements during treatment initiation or adjustment to fully determine its utility as a reliable monitor of therapeutic efficacy.

## 5 Conclusion

Nonetheless, present findings are primarily derived from limited retrospective and cross-sectional studies, which constitute a significant research gap regarding long-term prognostic power. Future research must, therefore, be explicitly directed toward initiating large-scale, multi-center, prospective trials to rigorously validate PIV’s clinical utility. These efforts are crucial to establish optimal, standardized cut-off values and to assess its true predictive power over time and across diverse RA populations. Ultimately, PIV represents a significant and practical biomarker that holds the potential to substantially improve the accuracy and personalization of RA management, offering clinicians a more dependable, affordable measure for assessing disease progression and treatment efficacy.
